# Antimicrobial Efficacy of Two Different Calcium Hydroxide Endodontic Dressings on the Eradication of Enterococcus faecalis in Single-Rooted Canals: An In Vitro Study

**DOI:** 10.7759/cureus.40056

**Published:** 2023-06-06

**Authors:** Paola G Rumhein, Kinda J Layous, Hassan Achour, Mudar Mohammad Mousa, Haya Deeb, Mohammad Y Hajeer

**Affiliations:** 1 Department of Endodontics and Restorative Dentistry, Faculty of Dentistry, University of Damascus, Damascus, SYR; 2 Department of Orthodontics, Faculty of Dentistry, University of Damascus, Damascus, SYR; 3 Department of Public Health, Faculty of Medicine, University of Damascus, Damascus, SYR

**Keywords:** calcium hydroxide paste, calcium hydroxide plus points (reoko), endodontic dressings, enterococcus faecalis, saline, single-rooted premolars

## Abstract

Introduction: Bacterial pulp infections are considered the most common cause of endodontic treatment failure. *Enterococcus faecalis* was isolated from most cases of failure of endodontic treatment. Therefore, using the appropriate intra-canal dressing is essential for successful treatment. The improved formula used in calcium hydroxide PLUS points ensures more calcium hydroxide is released over a longer period and more space to release calcium hydration. This in vitro research aimed to evaluate the differences in the efficacy between Ca(OH)_2_ paste and PLUS points as an endodontic dressing in eradicating *E. faecalis* growth inside infected single-rooted canals.

Materials and methods: Thirty mandibular first premolars with single canals were extracted for orthodontic reasons and were prepared after cutting their crowns to standardize the length of the roots to 17 mm, root preparation, and isolating *E. faecalis*. The infected sample root canals were contaminated with the prepared bacterial suspension, and the sample was incubated in the incubator under air conditions at 37°C for seven days, counting the bacteria colonies. Then, the bacterial units were counted before applying the drug, applying Ca(OH)_2_ paste in the first group and Ca(OH)_2_ PLUS points in the second group. The bacterial units were counted, and the number of bacteria was compared between the two substances applied to the samples, measuring the intracanal dressings' effectiveness. Wilcoxon signed-rank tests were used to detect significant differences.

Results: The results showed a statistically significant difference in the bacterial count of *E. faecalis* before and after applying the dressing of Ca(OH)_2 _paste from a mean of 11.89 to a mean of 3.18 (p=0.003) and no statistical difference in applying Ca(OH)_2_ PLUS points from mean 11.98 to mean 10.50 (p>0.05).

Conclusion: Within the limits of the current in vitro study, the Ca(OH)_2_ paste cones were more effective than Ca(OH)_2_ PLUS points in eradicating *E. faecalis* growth inside the infected single-rooted canals.

## Introduction

Bacterial pulp infections are considered the most common cause of endodontic treatment failure [1]. Microorganisms that remain in the apical parts of the root canal system are responsible for this failure, even if they seem to be well endodontically treated [1], particularly, *Enterococcus faecalis *that were insulated from most cases of failure of endodontic treatment [2]. *E. faecalis *are Gram-positive bacteria classified within the species of Streptococcus, and they are free non-aerial bacteria that can grow in the presence or absence of oxygen [3].

Various chemotherapy endodontic treatments are delivered to the apical region differently [4]. Therefore, using the appropriate intra-canal dressing has a major role in decreasing the microbial population within the root [5]. For optimal effect, calcium hydroxide Ca(OH)_2_ has been the gold standard for pulp intracanal dressings [6]. Nevertheless, the difficulty lies in applying it in the root canal toward the apex and removing it [7].

Due to its difficulties, an alternative for intra-canal dressing has developed to use Ca(OH)_2 _containing gutta-percha points with antibacterial activity (calcium hydroxide PLUS points cones) (ROEKO Gelatamp; Altstätten, Switzerland: COLTENE Group) [8]. Calcium hydroxide PLUS points cones are easily inserted directly into the canal along the entire working length, and adding a drop of sterilized water can accelerate its initial release. Reviewing the literature reveals that there is a lack of research done to evaluate the impact of the use of calcium hydroxide PLUS points (ROEKO) on eradicating *E. faecalis *when disinfecting root canals. Therefore, the objectives of the current in vitro study were to evaluate the effectiveness of Ca(OH)_2_ paste and PLUS points as an endodontic dressing in the eradication of *E. faecalis *growth inside infected single-rooted canals.

## Materials and methods

Study design and setting

This is an in vitro study. The Local Research Ethics Committee of the University of Damascus approved the research project to be conducted at the Faculty of Dentistry and the Faculty of Medicine (#UDDS-458-19122021/SRC-3654). In addition, signed informed consent was obtained from all participants for using their dental samples in the study. The postgraduate research budget of the University of Damascus was responsible for supporting the research work (ref no: 53173680DEN).

Sample collection

**Figure 1:**
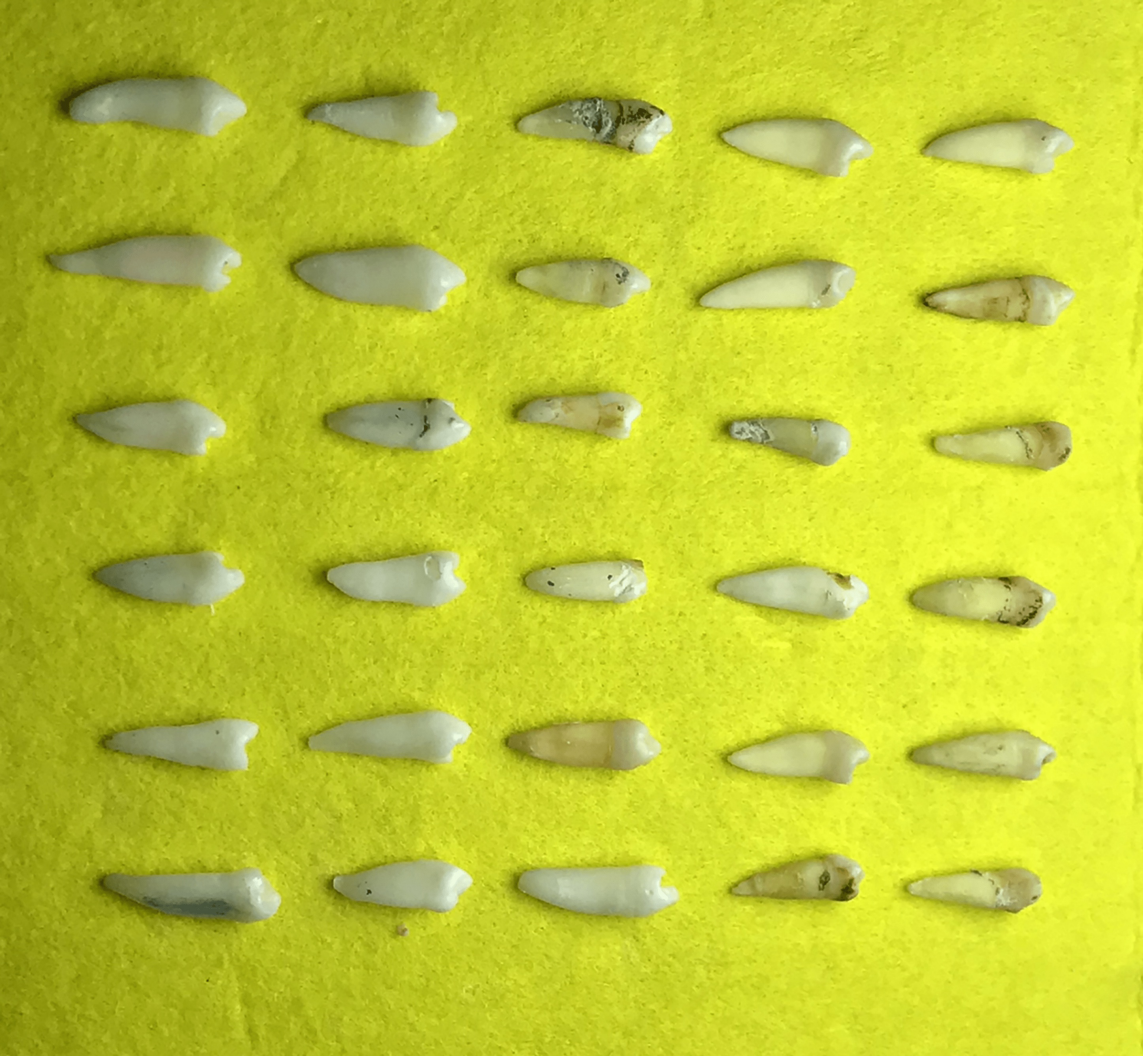
The complete sample of 30 extracted first lower premolars included in the current study.

Sixty premolars from 60 patients were selected from extracted human first lower premolars for orthodontic reasons at Endodontic Clinic, Faculty of Dentistry. Radiographs were taken by using a digital X-ray sensor EzSensor (Hwaseong, Korea: VATECH) to confirm the presence of single-canaled, single-root premolar with a complete apex as those features make the premolar easier and clearer to use. Additionally, the included first lower premolars should have been extracted for orthodontic reasons without any internal resorption, root cracks, anatomical changes, previous endodontic treatment, periodontic disease, calcifications, and pathological changes. Depending on the inclusion criteria, only 30 first lower premolars were included, and they were randomly distributed into two groups; group A included 15 premolars to receive the Ca(OH)_2_ paste (Al-fares, Damascus, Syria), whereas group B included 15 premolars to receive ROEKO Ca(OH)_2_ PLUS points (ROEKO Gelatamp; Altstätten, Switzerland: COLTENE Group) (Figure 1).

Root preparation

Root surface debridement was done by using U15 manual scaling tool (Ho Chi Minh City, Vietnam: Dentaluck), and then samples were immersed in a 5.25% sodium hypochlorite solution for one hour, then in sterilized physiological serum till preparation. Dental crowns were cut by diamond to the lengths of teeth to 17 mm; the root canals were expanded using 15-20 K-file (Tokyo, Japan: Mani Inc.), then the canals were prepared with Fanta F-One (Shanghai, China: FANTA) auto-preparation single file preparation system to the entire working length, then composites (Tetric N-Ceram; Schaan, Germany: Ivoclar) were applied to seal the apices of roots. After that, the roots were coated with two layers of red nail polish to prevent bacterial infiltration, and the roots were placed in cold acrylic molds. The samples were individually inserted into the sterilization bags in moist heat at a temperature of 121°C for 15 minutes, and a test was conducted to ensure the sterility of the teeth.

Bacteria isolation

**Figure 2:**
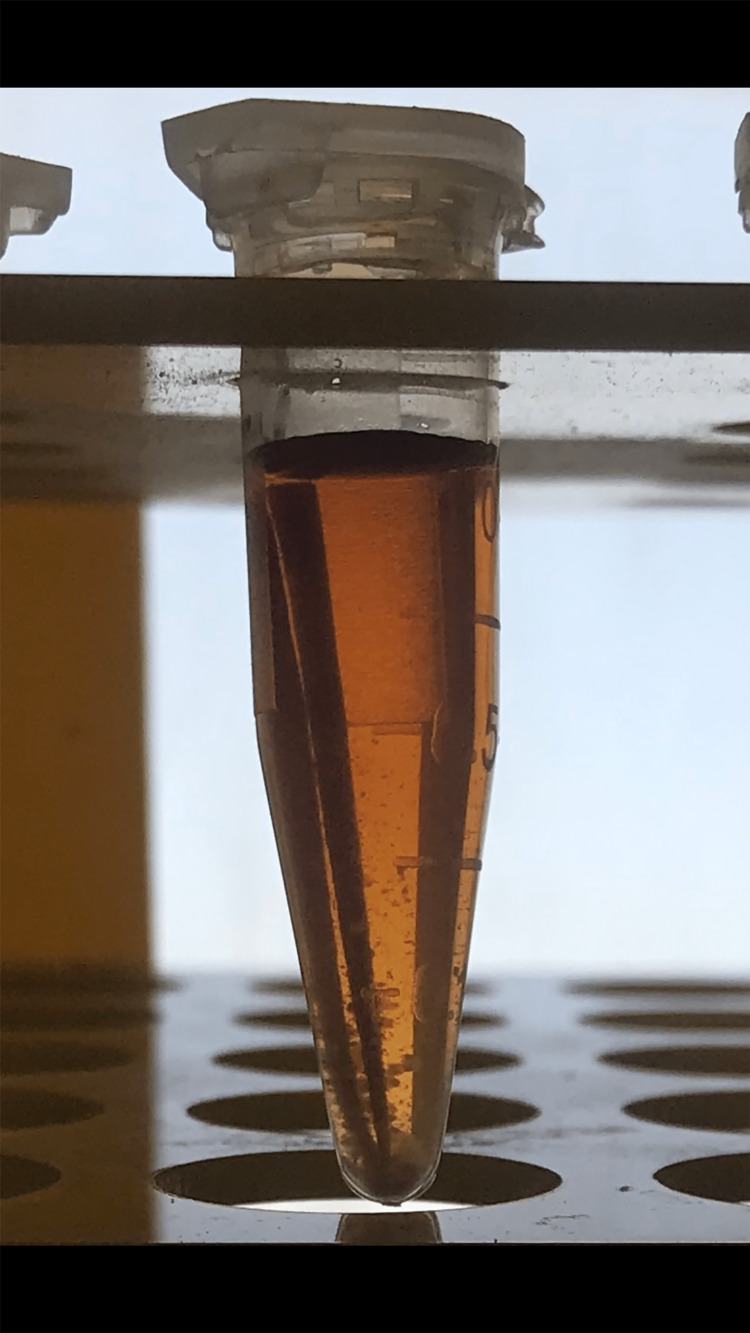
Tube containing a nutrient broth (the Brain Heart).

**Figure 3:**
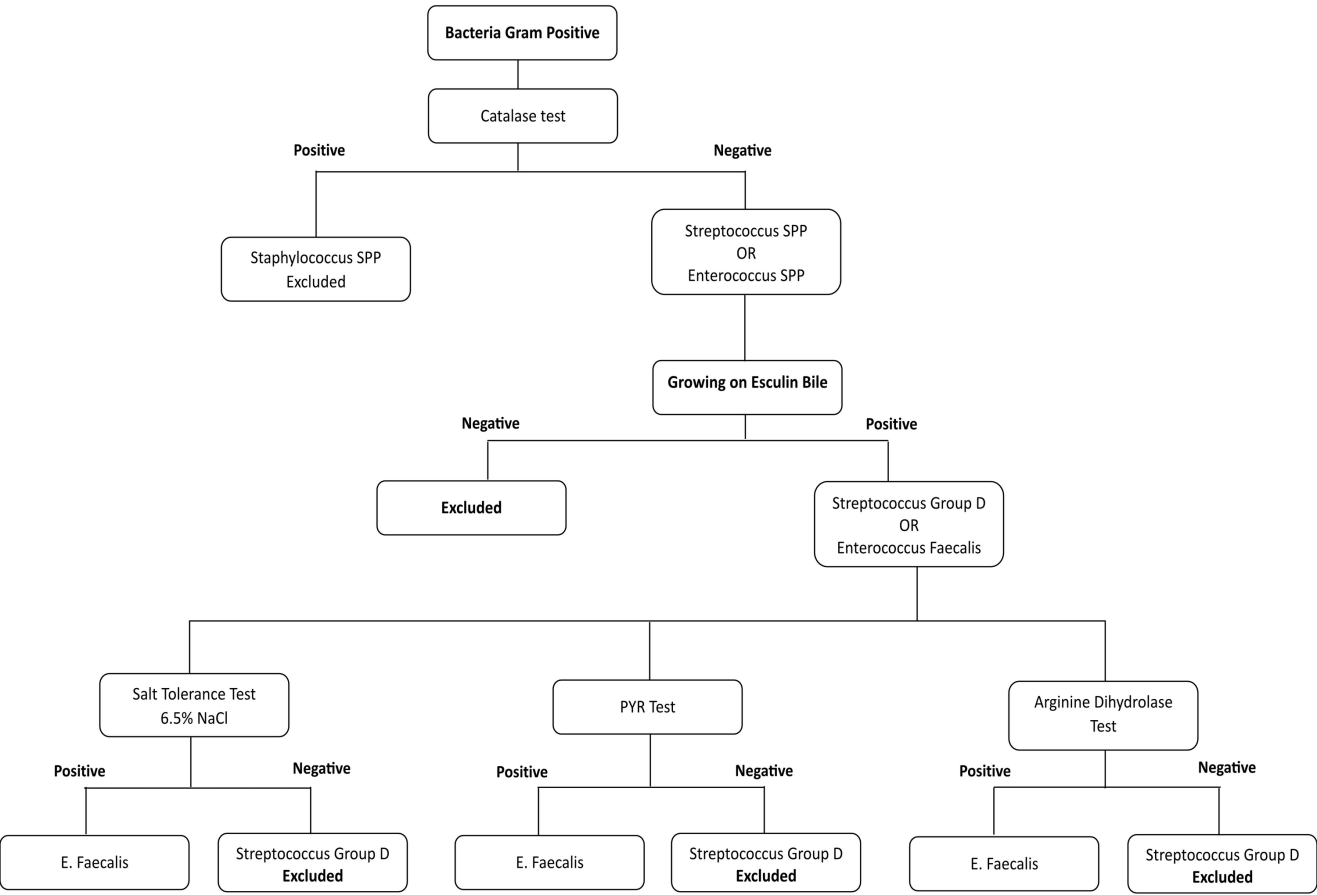
Several tests were conducted to isolate Enterococcus faecalis bacteria. PYR: pyrrolidonyl arylamidase

*E. faecalis *bacteria were clinically isolated, and samples were taken from infected root canals with chronic abscesses; then, two paper cones were inserted separately for 60 seconds into each cone. These cones were placed in a tube containing a nutrient broth (that nourished the Brain Heart), then the nutrient broth was taken directly and placed in the incubator for 24 hours at an aerial medium at 37°C (Figure 2). In the following step, a sample of cultured broth was taken, and the colonies were isolated on a general cultured medium blood agar and incubated in aerial conditions at 37°C for 24 hours. Several tests were conducted to isolate *E. faecalis *bacteria in the Laboratory of Medical Technical Institute at the Faculty of Medicine, University of Damascus (Figure 3).

**Figure 4:**
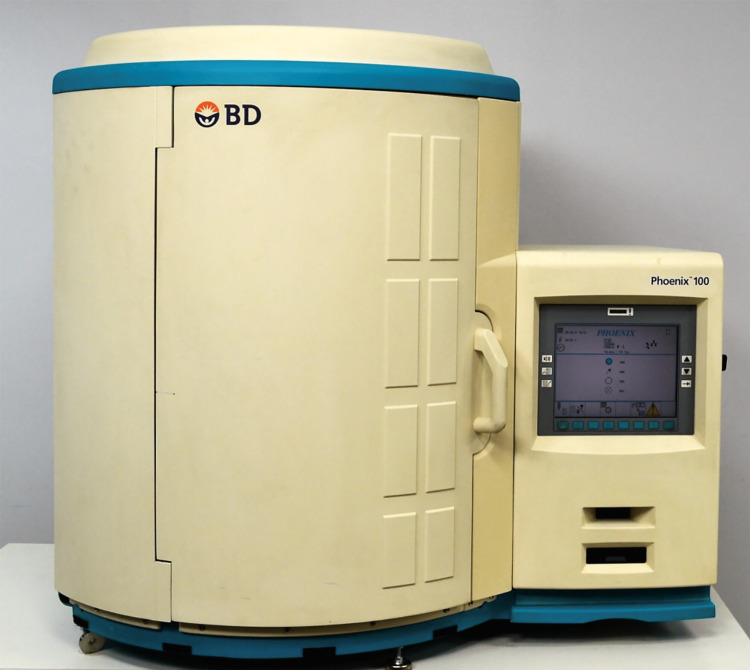
The machine Phoenix with an automated system.

Several colonies were taken and stained by Gram method, and the smear was examined under an optical microscope (Tokyo, Japan: Olympus Group) using a submersible lens after adding one drop of rice oil to distinguish the type of bacteria, Gram-negative or Gram-positive. Only Gram-positive cocci were taken, and they were used to determine whether they were *E. faecalis* by culturing them in bile esculin agar (BEA) (Berlin, Germany: Sifin) medium/test, conducting salinity tolerance test and arginine hydrolysis test. A sample was sent to the Laboratory of Al-Mouwasat University Hospital, University of Damascus to conduct phage typing of the bacteria on the machine Phoenix automated system (Heidelberg, Germany: Becton Dickinson) and to verify that it was *E. faecalis *(Figure 4).

Sample infection with *E. faecalis *bacteria

One load of platinum loops was taken from *E. faecalis *culture and put in 5 mL, and it was emulsified in a tube containing 5 mL of nutrient broth Brain Heart (Mumbai, India: HiMedia), then the tube was incubated for 24 hours at 37°C. Next, the teeth of the two groups were injected with a micropipette with an equal amount of 50 µ in each canal from the prepared bacterial suspension after adjusting the intensity to McFarland 0.5. Moreover, the nutrient broths inside the teeth were renewed during the incubation period to ensure that there was no dehydration of the intra-canal bacterial suspension.

Preliminary counting of the units forming bacterial colonies from the roots

**Figure 5:**
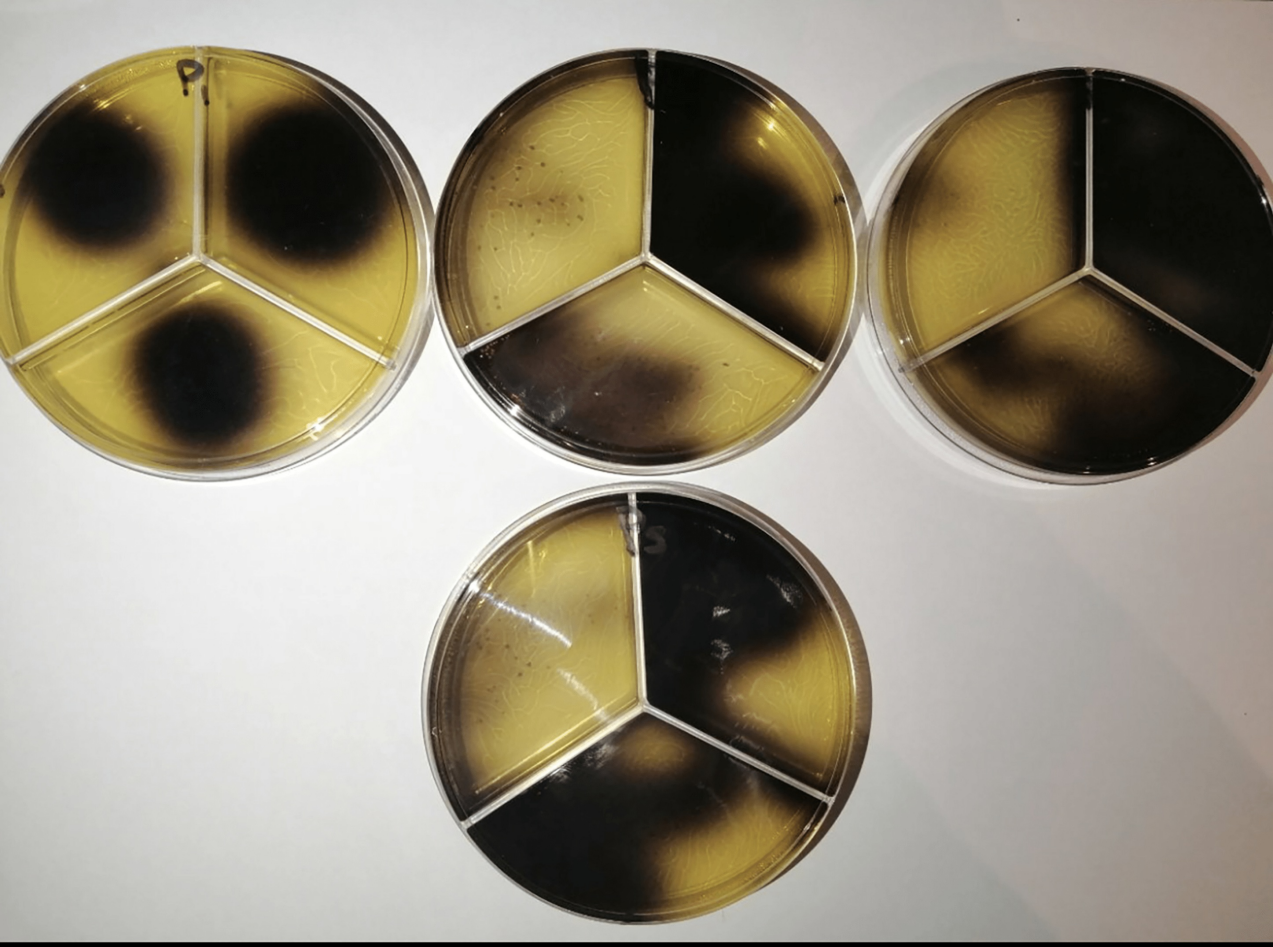
Preliminary counting of the units forming bacterial colonies from the roots.

The bacteria inside the root canals were counted by the viable count method as the canal was filled with the physiological serum, and the bacterial sample was taken in a red sterile paper cone - 25 sizes. Then, it was transferred to a tube containing 1 mL of physiological serum and mixed with the shaker for 20 seconds; then, 3 decimal extensions were conducted with continuous mixing for each tube with the shaker for 20 seconds. We aimed to approach an appropriate extension to count bacteria (Figure 5).

Application protocol of intra-canal dressings

In the first group, 1 g of calcium hydroxide paste was mixed with 1.5 mL of 0.9% saline solution. Then the canals were filled with the mixture. In the second group, calcium hydroxide PLUS points-saline from Coltene was applied to all 15 teeth. The canals were filled with cones after adding saline drops to the cone. In both groups, the coronal orifice of each root was sealed with wax. Then the samples were placed inside two sterilized Becher glasses, wrapped with tin foil, and placed into the incubator for seven days.

Counting of units forming bacterial colonies after dressing application

One week after applying the filler, the canal was filled with sterilized physiological serum, and the solution was left inside the canal for 15 seconds with conducting peripheral filing using an h-file, and bacterial swabs with 25-sized red paper cones were taken. The cone was transferred to a sterilized Eppendorf tube containing 1 mL of sterile saline solution, and again the swab was repeated three times for each canal to obtain the true bacterial reality. The tube containing the paper cones was shacked for one minute using a bio vortex device, and 50 µ of fluid was taken and cultured on a Petri dish divided into two parts containing esculin bile agar medium and the extension of the remaining fluid in an Eppendorf tube by placing it in a sterilized tube containing 9 mL of sterilize saline serum solution and shaking the tube for 1 minute. Fifty microns from the solution inside the tube were cultured on pre-prepared Petri dishes and placed in the incubator within special conditions. Twenty-four hours later, the dishes were taken out of the incubator, and the microbial units were counted by viewing using the counting device colony counter 560.

**Figure 6:**
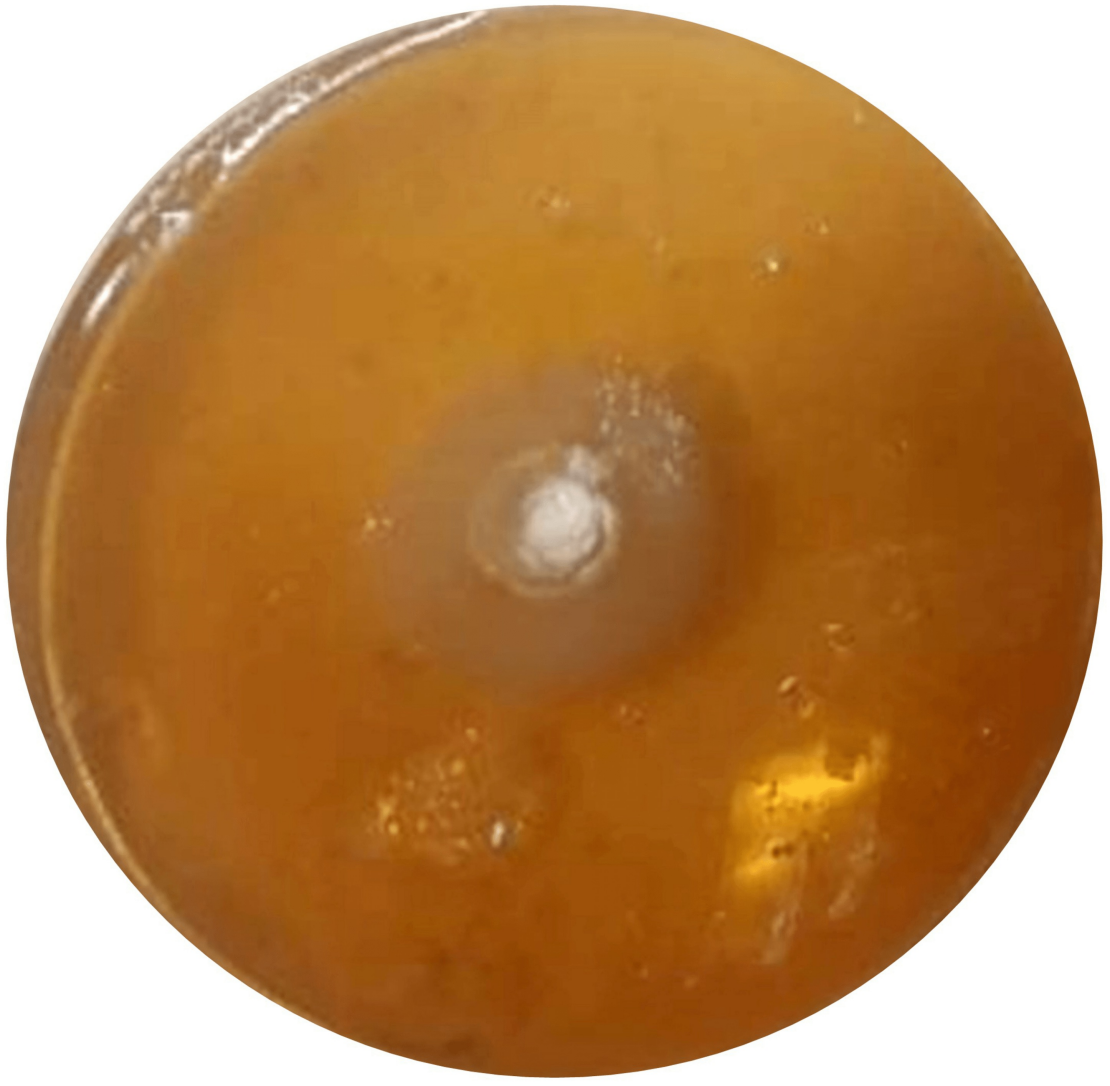
Calcium hydroxide paste had a full antimicrobial efficacy.

**Figure 7:**
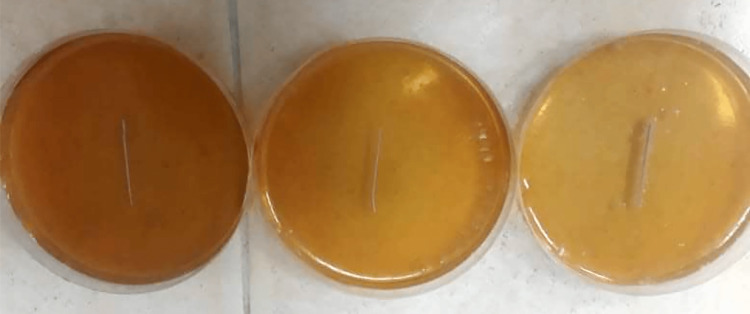
Calcium hydroxide PLUS points had no antimicrobial efficacy.

To confirm the work on the teeth directly, two Mueller-Hinton agar dishes were prepared for allergy study. One colony of *E. faecalis *was spread on the two dishes after being taken from a blood-agar dish incubated at 37°C for 24 hours, and the density was adjusted to 0.5 McFarland. Ca(OH)_2_ paste was placed on the first dish, and Ca(OH)_2_ PLUS points on the second. The results are read by measuring the aura formed around the dressings; they indicated that Ca(OH)_2_ paste had a full antimicrobial efficacy (Figure 6) and Ca(OH)_2_ PLUS points had no efficacy in reducing the bacterial growth count of *E. faecalis* (Figure 7).

Statistical analysis

The statistical analysis was made using the Statistical Package for Social Sciences version 24 (Chicago, IL: SPSS Inc.). Microsoft Excel program was used in collecting and clarifying the results of counted microbial units. The Shapiro-Wilk test was used to determine the normality of the distributions. The test showed that the data was not normally distributed in either group, i.e., the Ca(OH)_2_ paste (CH group) and Ca(OH)_2_ PLUS points (CH+P group) (p=0.09 and 0.93, respectively). Therefore, Wilcoxon signed-rank matched-pairs tests were used to detect differences within each group. The level of significance was set at 5%.

## Results

**Figure 8:**
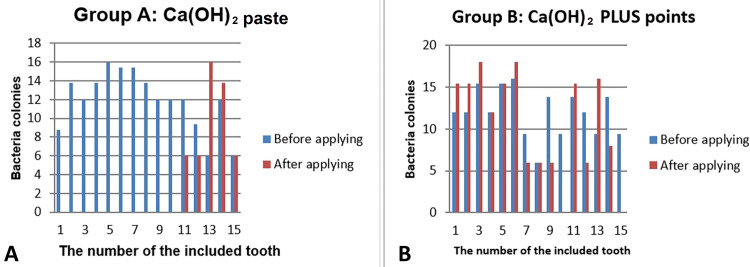
Bar chart showing the counts of bacterial growth before and after applying the intra-canal dressings. The images show (A) calcium hydroxide paste group and (B) calcium hydroxide PLUS points.

The descriptive statistics of bacterial growth colonies before and after applying the calcium hydroxide paste in the CH group and calcium hydroxide PLUS points in the CH+P group are given in Table 1, and the related charts are shown in Figure 8.

**Table 1:** Descriptive statistics of bacteria count in the canals before and after applying the intra-canal dressings in each group.

Tooth ID	Group A: Ca(OH)_2_ paste (n=15)		Group B: Ca(OH)_2_ PLUS points (n=15)	
	Before applying (n=15)	After applying (n=15)	Before applying (n=15)	After applying (n=15)
1	8.8	0.0	12.0	15.4
2	13.8	0.0	12.0	15.4
3	12.0	0.0	15.4	18.0
4	13.8	0.0	12.0	12.0
5	16.0	0.0	15.4	15.4
6	15.4	0.0	16.0	18.0
7	15.4	0.0	9.4	6.0
8	13.8	0.0	6.0	6.0
9	12.0	0.0	13.8	6.0
10	12.0	0.0	9.4	0.0
11	12.0	6.0	13.8	15.4
12	9.4	6.0	12.0	6.0
13	6.0	16.0	9.4	16.0
14	12.0	13.8	13.8	8.0
15	6.0	6.0	9.4	0.0
Range	6.0-16.0	0.0-16.0	6.0-16.0	0.0-18.0
Mean (±SD)	11.89 (±3.1)	3.18 (±5.3)	11.98 (±2.7)	10.50 (±6.1)
Median	12.0	0.0	12.0	12

The mean of the growing bacteria in the CH group was 11.89±3.1 CFU/mL, with a range between 6.0 CFU/mL and 16.0 CFU/mL. The CH+P group's mean was closer to that of the CH group; 11.98±2.73 CFU/mL, with a range between 6.0 CFU/mL and 16.0 CFU/mL. However, the mean of the growing bacteria after applying Ca(OH)_2_ paste became 3.18±5.3 CFU/mL, and the mean of the CH+P group became 10.50±6.1 CFU/mL after applying Ca(OH)_2_ PLUS points. The CH group showed a statistically significant difference in the counts of *E. faecalis *bacteria before and after applying the dressing (from 11.89 to 3.18, p=0.003) (Table 2). In the CH+P group, there is no statistically significant difference in the count of *E. faecalis *bacteria before and after applying the dressing (from 11.98 to 10.50, p=0.293).

**Table 2:** Inferential statistics regarding the differences in the bacterial count before and after applying the two types of intra-canal dressings. *P-value calculated using Wilcoxon matched-pairs signed-rank test. CH: calcium hydroxide paste group; CH+P: calcium hydroxide PLUS points group

Group	Assessment time	N	Median	Mean	p-Value*
CH	Before	15	12.0	11.89	0.003
	After	15	0.0	3.18	
CH+P	Before	15	12.0	11.98	0.293
	After	15	12.0	10.50	

By comparing the efficacy rate in inhibiting the bacterial growth between the two intra-canal dressings, the efficacy rate of the CH group was 66.6% (10 out of 15), and the efficacy rate in the CH+P group was 13.3% (two out of 15). In other words, Ca(OH)_2_ paste was five times more effective in inhibiting the *E. faecalis *growth inside the infected canals.

## Discussion

This study evaluated the antimicrobial effect of two different types of Ca(OH)_2_ intracanal dressing on reducing *E. faecalis *growth in the infected canals. The reason for the seriousness of this bacterium is its ability to invade the dentinal canals [9,10], causing, in an unusual way, persistent apical periodontitis [11], and it is very resistant to eliminate from the root canals [12].

For optimal effect, the endodontic treatment could be summed up in three steps: mechanochemical preparation, controlling the growth, and reducing the bacteria count; the root canal was well-filled. We, as dentists, can control each part of this endodontic treatment process except the efficacy of the used substance in reducing the bacterial count in the canal. Among the dressings commonly used in endodontic treatments are the Ca(OH)_2_ paste dressing, which was first suggested in 1920 by Hermann, as mentioned by Ma et al. [13]. However, the difficulty in removing this dressing from the root canals is well-known in the literature [13]. On the other hand, modern research worked to develop gutta-percha cones loaded with calcium hydroxide and chlorhexidine, which can be placed in the root canal and removed from it easily [14].

This experiment showed that Ca(OH)_2_ paste was very effective in eradicating *E. faecalis *growth inside infected single-rooted canals, in contrast to Ca(OH)_2_ PLUS points. The inefficacy of Ca(OH)_2_ PLUS points in this experiment conformed with other bacterial laboratory studies, which showed the inability of gutta-percha with calcium hydroxide to inhibit the growth of several bacteria, including *E. faecalis *[15]. Some researchers also confirmed that the calcium hydroxide PLUS points did not produce any sterile bacterial cultures even if it reduced bacterial growth [16-18].

Otherwise, Ca(OH)_2_ paste has an effective antimicrobial activity in almost all the previous experiments [19-21], and it was found that the higher the concentration of the paste, the greater the inhibition ability [22]. Furthermore, using Ca(OH)_2_ paste and PLUS points in a direct way on a cultured *E. faecalis *plate indicated the ineffectiveness of Ca(OH)_2_ PLUS points compared to Ca(OH)_2_ paste. This finding is in agreement with another study that used a similar direct way of measuring the antimicrobial activity of Ca(OH)_2_ paste and Ca(OH)_2_ points compared to saline as a control but by using a tube dilution test [23].

Although the small number of sample is considered the most important limitation of our research, the results of the effectiveness of calcium hydroxide PLUS points have not yet been indicated. Despite its ease of use and modernity, calcium hydroxide paste still has a better antimicrobial activity as an intracanal dressing. Therefore, we highly recommend further research on the efficacy of Ca(OH)_2_ PLUS points, either in vivo or in vitro experiments.

## Conclusions

The success of the endodontic treatment is mainly related to the appropriate intracanal dressing used in the treatment and its antimicrobial activity, especially against infected canals with *E. faecalis *bacteria. This in vitro study indicated that despite the ease of using Ca(OH)_2_ PLUS points, it did not have any antimicrobial activity against *E. faecalis *by comparing it to Ca(OH)_2_ paste which showed a 66.6% efficacy rate in inhibiting bacterial growth.
