# Correction: Antimicrobial Efficacy of Two Different Calcium Hydroxide Endodontic Dressings on the Eradication of Enterococcus faecalis in Single-Rooted Canals: An In Vitro Study

**DOI:** 10.7759/cureus.c422

**Published:** 2026-04-09

**Authors:** Paola G Rumhein, Kinda J Layous, Hassan Achour, Mudar Mohammad Mousa, Haya Deeb, Mohammad Y Hajeer

**Affiliations:** 1 Department of Endodontics and Restorative Dentistry, Faculty of Dentistry, University of Damascus, Damascus, SYR; 2 Department of Orthodontics, Faculty of Dentistry, University of Damascus, Damascus, SYR; 3 Department of Public Health, Faculty of Medicine, University of Damascus, Damascus, SYR

This article has been corrected to fix a typo in Figure 3 where "positive" and "negative" were erroneously transposed beneath "Growing on Esculine Bile". The image has now been replaced with the correct version.

